# P-773. *Mycobacterium Tuberculosis* Liver Abscess in Kidney Transplant Recipients

**DOI:** 10.1093/ofid/ofae631.968

**Published:** 2025-01-29

**Authors:** Natasha Dyal, Holenarasipur R Vikram, Thilagavathi Venkatachalam, Lavanya Kodali

**Affiliations:** Mayo Clinic, Scottsdale, Arizona; Mayo Clinic, Scottsdale, Arizona; Mayo Clinic, Scottsdale, Arizona; Mayo Clinic, Scottsdale, Arizona

## Abstract

**Background:**

Recipients of solid organ transplant (SOT) have higher risk for infections, and the prevalence of *mycobacterium tuberculosis* liver abscess (MTB-LA) without pulmonary/disseminated MTB is 0.34%. However, there is little research on MTB-LA in SOT recipients.

Mycobacterium tuberculosis Liver Abscess Cases

**Figure:**
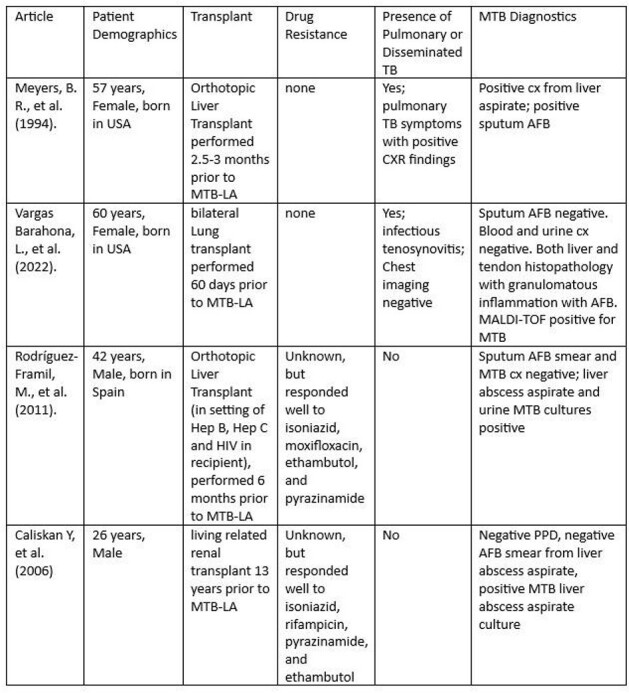

Upon extensive literature review, this chart details all published, English-language cases of mycobacterium tuberculosis liver abscess in solid organ transplant recipients.

**Methods:**

We conducted an extensive literature review of MTB-LA in adult SOT recipients.

**Results:**

Our review showed 4 cases of MTB-LA in SOT recipients (table below). We detail 1 additional case.

A 58-year-old male with ESRD due to type 2 diabetes mellitus, and latent MTB underwent kidney transplantation in March 2023. He completed a 6-month course of isoniazid and vitamin B6 for latent MTB 1 month before transplant. His post-operative course was uncomplicated; he was discharged to follow up with his nephrologist. He later developed norovirus illness with fever, which was conservatively managed. He developed new fevers 3 months post-transplant and CT of the abdomen/pelvis revealed a liver abscess, which was treated with empiric broad spectrum antibiotics, and was not amenable to aspiration. At his 4-month post-transplantation, he complained of fatigue, night sweats, poor appetite, and weight loss requiring hospitalization, wherein an MRI of the abdomen/pelvis identified a 3.6 x 3.2 x 6.2 cm liver abscess. The liver abscess aspirate was positive on acid fast bacilli smear and MTB polymerase chain reaction. He was initiated on antituberculosis therapy with isoniazid, rifabutin, pyrazinamide, and ethambutol. Susceptibilities identified pyrazinamide resistance, and led to substitution with levofloxacin. Labs, imaging and microbiology testing were negative for evidence of pulmonary/disseminated MTB. His donor’s information was reviewed; no obvious risk factors for active MTB were found. The recipient of the mate kidney is doing well without evidence of MTB infection. After 2 months of 4-drug antituberculosis therapy, ethambutol was discontinued. CT scan of the abdomen and pelvis at 4 months post anti-tuberculosus therapy revealed a 3 cm area of residual inflammation at the site of prior MTB-LA, in the setting of resolution of prior symptoms.

**Conclusion:**

Early imaging and diagnostic aspiration of liver abscess with specific stains, culture and PCR testing can facilitate rapid diagnosis and initiation of antituberculosis therapy.

**Disclosures:**

**All Authors**: No reported disclosures

